# Enhancing Optical Correlation Decision Performance for Face Recognition by Using a Nonparametric Kernel Smoothing Classification

**DOI:** 10.3390/s19235092

**Published:** 2019-11-21

**Authors:** Matthieu Saumard, Marwa Elbouz, Michaël Aron, Ayman Alfalou, Christian Brosseau

**Affiliations:** 1 Yncrea Ouest, Artificial Intelligence and Emerging data Laboratory, 2 rue de la châtaigneraie, 35510 Cesson-Sevigné, France; michael.aron@isen-ouest.yncrea.com; 2 Yncrea Ouest, Artificial Intelligence and Emerging data Laboratory, 20 rue du Cuirassé Bretagne, 29200 Brest, France; marwa.el-bouz@isen-ouest.yncrea.fr (M.E.); ayman.al-falou@isen-ouest.yncrea.fr (A.A.); 3 Univ Brest, CNRS, Lab-STICC, 6 avenue Le Gorgeu, 29238 Brest Cedex 3, France; brosseau@univ-brest.fr

**Keywords:** face verification, optical correlation, Hausdorff distance, image classification

## Abstract

Optical correlation has a rich history in image recognition applications from a database. In practice, it is simple to implement optically using two lenses or numerically using two Fourier transforms. Even if correlation is a reliable method for image recognition, it may jeopardize decision making according to the location, height, and shape of the correlation peak within the correlation plane. Additionally, correlation is very sensitive to image rotation and scale. To overcome these issues, in this study, we propose a method of nonparametric modelling of the correlation plane. Our method is based on a kernel estimation of the regression function used to classify the individual images in the correlation plane. The basic idea is to improve the decision by taking into consideration the energy shape and distribution in the correlation plane. The method relies on the calculation of the Hausdorff distance between the target correlation plane (of the image to recognize) and the correlation planes obtained from the database (the correlation planes computed from the database images). Our method is tested for a face recognition application using the Pointing Head Pose Image Database (PHPID) database. Overall, the results demonstrate good performances of this method compared to competitive methods in terms of good detection and very low false alarm rates.

## 1. Introduction

The use of correlation methods [1,2,3,4] remains very competitive despite the abundance of purely numerical methods, such as Support Vector Machines and neural networks. Correlation is easy to use in practice because it is based on two Fourier transforms (FTs) and one multiplication in the frequency domain [5].

For comparison, a deep learning-based method has generally good performance but also significant drawbacks due to algorithm complexity, implementation difficulty, time-consuming learning processes, and a high number of computational resources [6]. Most of the developments are devoted to increasing the performance of correlation methods concentrated in the Fourier plane [7,8,9,10,11] by designing innovative correlation filters. On the other hand, there exists a growing scientific community dealing with biometric issues, such as face recognition, fingerprint detection, and early automatic disease detection [12,13]. The primary focus of this paper is to deal with an authentication problem using a database. There are two kinds of issue, i.e., identification and verification. Here, our primary goal is to optimize the solution to verification.

In order to improve the decision performance, our model uses a statistical learning method, i.e., a supervised classification method. The regression function between the binary output (class of person) and the input (correlation plane) is nonparametrically estimated for the learning database by making use of the modified kernel smoothing Nadaraya–Watson algorithm [13,14].

Functional data analysis has recently been developed to statistically analyze curves or objects, see e.g., Reference [15] for a good introduction to this subject. The interested reader may also refer to [16] for an overview of nonparametric estimation with functional data. In [17], the authors defined an extension of the Nadaraya–Watson estimator for objects such as curves by introducing a distance in the kernel between two functional objects. Here, we propose the use of kernel smoothing estimation to cope with the correlation plane, and we choose an appropriate distance, i.e., the Hausdorff distance, to plug in the kernel for estimating the regression function. As a result, it is possible to propose a decision-making protocol which has dual effects for increasing good decision rates and reducing false alarm rates.

The rest of this paper is organized as follows. Section 2 provides the correlation principle. After a short description of the database in Section 3, our overall method is explained in Section 4. Our model is implemented in Section 5. The method’s accuracy is checked in Section 6, which provides two series of simulation studies. Section 7 briefly concludes.

## 2. Modeling Correlation

**Figure 1:**
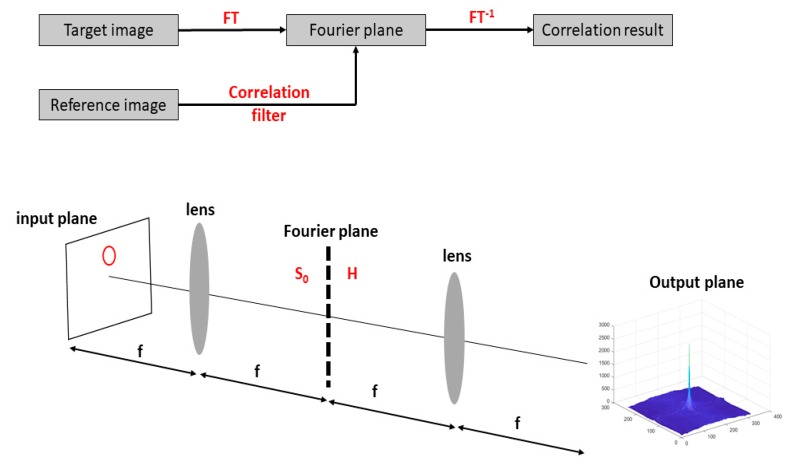
Illustrating the Vander Lugt Correlator (VLC) principle.

In essence, a Vander Lugt Correlator (VLC) compared a target image (input plane) with a reference image. The result of this comparison is presented in the form of a correlation plane. More precisely, the spectrum of a target image was obtained with a FT and was multiplied by a correlation filter made from the reference image [1,2,3,4,5]. An inverse FT (FT^−1^) was then applied to get the output plane containing a noised correlation peak. The measure of the highest peak (i.e., the peak-to-correlation energy (PCE)) characterized the similarities between the reference and the target images. To validate our approach, we used a classical phase-only filter (POF), see Figure 1.

## 3. Dataset

**Figure 2:**
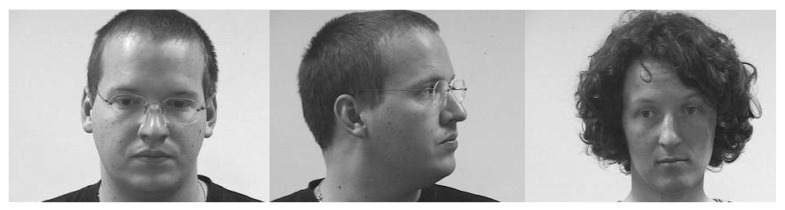
Three selected images from the Pointing Head Pose Image Database (PHPID) dataset.

Simulation results were obtained using the Pointing Head Pose Image Database (PHPID) [18]. This dataset includes 1302 face pictures: 14 different persons (93 images per person) with different orientations (from −90° to +90° with respect to the horizontal direction and from −10° and +10° with respect to the vertical direction). The resolution of each image is 314 × 238 pixels. It is also worth noting that this database includes a variety of persons (Figure 2), e.g., various skin colors, person with glasses or not, etc.

In this numerical study, different training/testing databases from the PHPID dataset were chosen in order to demonstrate the efficiency of our method. Once the specific dataset was chosen, tests were performed as follows: Firstly, a person from the testing database was chosen as a reference person (person 0). A classical POF filter and an autocorrelation plane were computed for each person in this dataset. The correlation technique, whose principle is shown in Figure 1, was applied to get the output correlation plane using the corresponding POF filter for each person in the database. A classification algorithm based on the Hausdorff distance was then used (Section 4). If the person was recognized, the analysis ended. If the person was not recognized, the procedure was repeated with another person from the training database until the training database was empty. Thus, only two possibilities exist, i.e., either the person is recognized or not.

**Figure 3:**
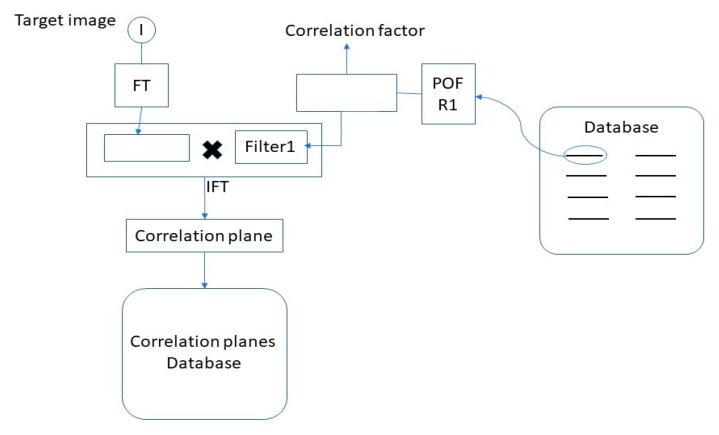
Flowchart illustrating the transition between the database and the correlation planes database.

Several remarks are in order, concerning Figure 3, which is organized in two parts. Part 1 defines the VLC, whose output is a plane of correlation. Part 2 is the decision part. For each face of the database, the VLC is used with the target image, thus resulting in a large collection of correlation planes. Next, the decision-making procedure relies on the Hausdorff distance between the target correlation plane and the correlation planes coming from the database by selecting a specific bandwidth (hereinafter described in Section 4).

By adopting a kernel smoothing classification algorithm, we took care of the shape, location, and denoising of the peak of correlation. From this algorithm, we learned which correlation plane was good, and we filtered bad correlation planes. The result of the algorithm was the variable $\hat{Y}$, the value of which is 0 for non-recognition decision, i.e., the person is not in the database and is equal to 1 for recognition, i.e., the person is in the database.

## 4. Overview of the Method

**Figure 4:**
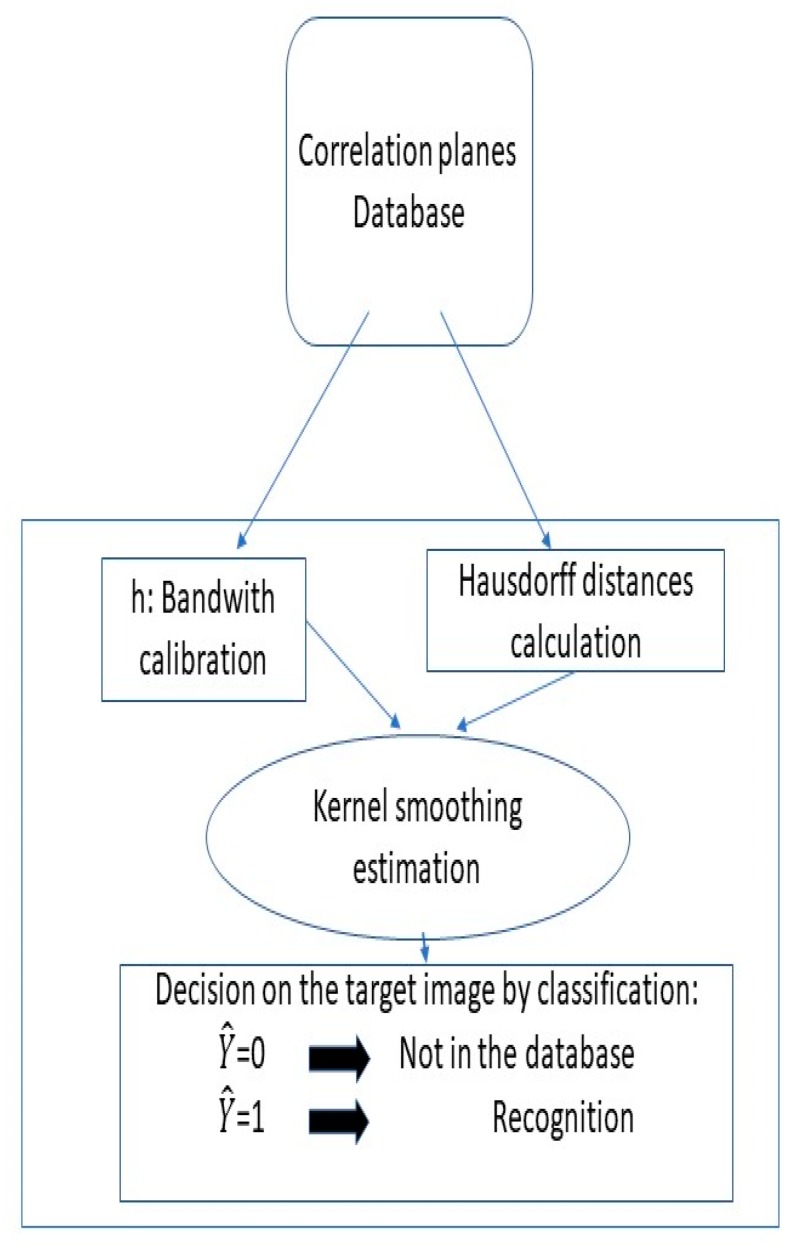
Flowchart illustrating the decision part.

**Figure 5:**
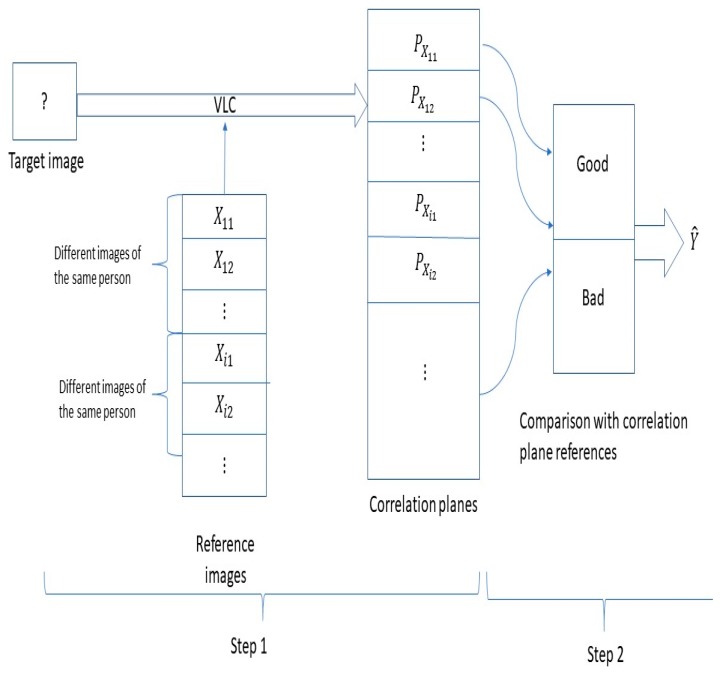
Flowchart illustrating the method.

The method was organized in two parts, see Figure 3, Figure 4 and Figure 5. The first part (Figure 3) was the construction of a new database containing computed correlation planes. This part was realized by calculating the correlation plane corresponding to a reference person (person 0) and all or a part of the series 1 of the PHPID dataset. The different ways of dividing the series 1 constitute the different training sets, which will be described in Section 6. The testing set is either the whole series 1 or the whole series 2.

The second part of our method was the decision part (Figure 4). Considering the correlation planes and the corresponding label indicating if they are the same person or not (in the training set), we made our decision on the testing set by estimating the probability of recognition via a kernel smoothing procedure.

Let us comment on the two-step algorithm represented in Figure 5.

Step 1: Step 1 began with the target image, which is the image to be recognized. The target image was introduced in a VLC correlator to be compared with all reference images of a database. This reference image database contains n different persons (X1,…, Xn) (Xi is the ith person); Xij represents the different variations that the person Xi can have (m is the number of variations considered).
  A set of reference images were used with the target image to generate different correlation planes (Px11,…,Pxnm). These correlation planes were then compared in step 2 with pre-computed correlation planes, known as the reference database.
Step 2: The correlation planes (Px11,…,Pxnm) were compared with a correlation plane database realized in step 1. This database was divided in two parts: The first part contained the good correlation plane of references and another part listed the bad correlations, i.e., the false correlation plane of references. We will compare the good and bad correlation planes in Section 5. The construction of these correlation planes of references was made as follows: The correlation planes of various images of person A, the correlation planes of various images of person B, …, and the correlations planes of various images of person Z, which constitute the good correlation planes of reference. The reference database of bad correlation planes was constructed as follows: We calculated the correlation planes between various images of person A and various images of person B, etc.

The comparison shown in Figure 5 was then realized with the Hausdorff distance and by making use of the kernel smoothing method, which realizes an estimation of the probability of belonging to the class of a known person (Section 5).

## 5. Nonparametric Model

The Hausdorff distance is widely used in the context of image recognition, see e.g., [19,20,21] for reviews. A modified version of the Hausdorff distance has been also applied to matching objects [22]. The Hausdorff distance can be defined as follows: Let E and F be two non-empty subsets of a metric space (*M*, *d*). The Hausdorff distance is given as:

$$d_{H}\left(E,F\right)=\max\{\;\underset{x\in E}{\sup}\;\underset{y\in F}{inf}\;d\left(x,y\right),\;\underset{x\in F}{\sup}\;\underset{y\in E}{inf}\;d\left(x,y\right)\}.$$

Here, for the purpose of comparing the target and database correlation planes, the Hausdorff distance between two planes (one with the unknown image and the other one calculated beforehand for the image present in the database) was evaluated. Once the distance was known, a nonparametric classification for decision making was performed.

Next, the decision part illustrated in Figure 4 is described. A kernel smoothing estimate of the regression function was employed. This estimate was used to perform a classification with a given threshold set to 0.5. Here, we made use of the Nadaraya–Watson estimator of the regression function [15] for classification. The principle is described as follows: Assume we have $\left(Y_{1},X_{1\;}\right),\ldots \;,\;\left(Y_{n},X_{n}\right)$ independent and identically distributed (i.i.d.) random variables coming from (Y,X) where *Y* is the variable labeled by 1, if the person is detected, and 0 otherwise, *X* is the corresponding correlation plane, and *n* denotes the sample size. Let us comment briefly on this i.i.d. sample: This collection of correlation planes was obtained from the learning database with person 0. Considering a new face image from the testing dataset, we computed the correlation plane with person 0. We then performed a nonparametric classification with a kernel estimate of the regression function E(Y|X). Assuming that Y is a Bernoulli random variable, then P (Y=1| X=x)=E(Y|X=x), where P represents the probability measure. Thus, P (Y=1| X=x) is the probability of detection, knowing the autocorrelation plane *x*. E(Y|X=x) is the expected value of Y, knowing the autocorrelation plane x. Now let us define an estimator of this probability as:

(1) $$\hat{Y}\;=\{\begin{array}{c} \frac{\sum_{i=1}^{n}Y_{i}K_{h}\left(d\left(x,X_{i}\right)\right)}{\sum_{i=1}^{n}K_{h}\left(d\left(x,X_{i}\right)\right)}\\ 0\;if\;\overset{n}{\underset{i=1}{\sum }}K_{h}\left(d\left(x,X_{i}\right)\right)=0 \end{array}\;$$

where $\hat{Y}$ is a prediction of Y, knowing the correlation plane x, keeping in mind that in our case it is also an estimate of the probability that Y is equal to 1 at the correlation plane x. Knowing $\hat{Y}$, we can decide if the face image is identical or not. If $\hat{Y}$ is close to 1, there is a high probability that there is a good match between the two persons, and if $\hat{Y}$ is close to zero, the probability that the two persons are not the same is large. In Equation (1), *K* is a real asymmetrical kernel, *h* is the bandwidth (calibration parameter), $K_{h}\left(.\right)=\frac{1}{h}K(\frac{.}{h})$, and *d* is the Hausdorff distance between images.

For the asymmetrical kernel, we used the asymmetrical version of the Epanechnikov kernel, namely $K\left(x\right)=\frac{3}{2}\left(1-x^{2}\right)1_{\left[0,1\right)}\left(x\right)$, where $1_{\left[0,1\right)}\left(.\right)$ stands for the indicator function on the set [0,1). The use of an asymmetrical kernel is standard in functional data analysis (see Ferraty and Vieu [17]) because the distance is positive for all planes of correlation. Other type of kernels can be used, but our numerical results show that the Epanechnikov kernel performs better than others. From Equation (1), we also see that the value of $\hat{Y}$ ranges from 0 to 1 and represents an estimation of the probability of Y to be of class 1, knowing that the X is at the target point x: P (Y= 1| X=x). Assume that $Y_{i’}$ is 1 and *x* is close to $X_{i’}$, then the procedure in Equation (1) will affect a value close to 1 to $\hat{Y}$.

For convenience, a threshold is set to 0.5, i.e., all values of $\hat{Y}$ larger to this threshold are recognized, and vice versa. This threshold value corresponds to an estimation of the Bayes classifiers [23]. The Bayes classifiers maximizes the probability P (Y=1| X=x). In our case, it corresponds to set $\hat{Y}$ = 1 to the plane of correlation with $\hat{Y}\;\geq \frac{1}{2}$ and $\hat{Y\;}$= 0 to $\hat{Y}<\frac{1}{2}$. In the next section, we illustrate this approach by considering two series of faces from the PHPID database.

## 6. Numerical Results and Discussion

### 6.1. A Brief Description of the Training Testing Set

Before the study, we needed correlation planes references. The good and bad planes of references were made like it is described in step 2 of Section 4. There are also training and testing sets. In order to clarify these notions, we refer to Figure 5. We call the training set the reference images of step 1 in Figure 5. The testing set represents the set of all the images of the target images.

### 6.2. Bandwidth Calibration

To achieve a good prediction, we must first find an optimal bandwidth. In other words, the parameter h, which appears in the Equation (1) via the kernel function, must be adjusted in order to eliminate bad behavior of our classification procedure. With the above assumptions in mind, this requires a tradeoff between bias and variance. A value of h close to 0 will give a good estimate of the regression function in the learning database. Otherwise, large values of h will eventually affect the overall error. The optimal bandwidth $\hat{h}$ realized this task: This is a good compromise between a low error term and a good capability of prediction. For this purpose, we used a leave-one-out cross validation procedure to estimate the bandwidth. The training set is made of 126 images: 9 deal with person 0. The optimal bandwidth is calculated as $h=argmin\;\overset{n}{\underset{i=1}{\sum }}{\left(Y_{i}-{\hat{Y}}^{\left(i\right)}\right)}^{2}$, where ${\hat{Y}}^{\left(i\right)}$ is the prediction for person i calculated without the i-th observation. We find that the optimal bandwidth $\hat{h}$ is 1.06. Now that the optimal bandwidth $\hat{h}$ is found, we can use the classification algorithm by fixing the value of $\hat{h}$ into Equation (1).

### 6.3. Simulations for a First Series of Faces from the PHPID Database

With a face image of person 0 chosen beforehand, our goal is to determine if person 0 belongs to a database or not by making use of our classification procedure. The first training set we considered was made of 126 images, where 9 were coming from person 0. We found that when using this training set, a mean square error (MSE) of 4.8% on the whole testing set (541 planes of correlation), corresponding to the first series of the PHPID database, and only one false positive, i.e., a false positive that is an error of prediction when the person must not be recognized, but the person is recognized by the algorithm as person 0, it is a type I error. The false negative is of a type II error, occurring when the result of the algorithm is negative while the true response must be positive. We found that 13 out of 39 images from the person of reference were recognized. If the entire database was used, the MSE was 0.92%, and only 5 images from person 0 were not recognized.

**Figure 6:**
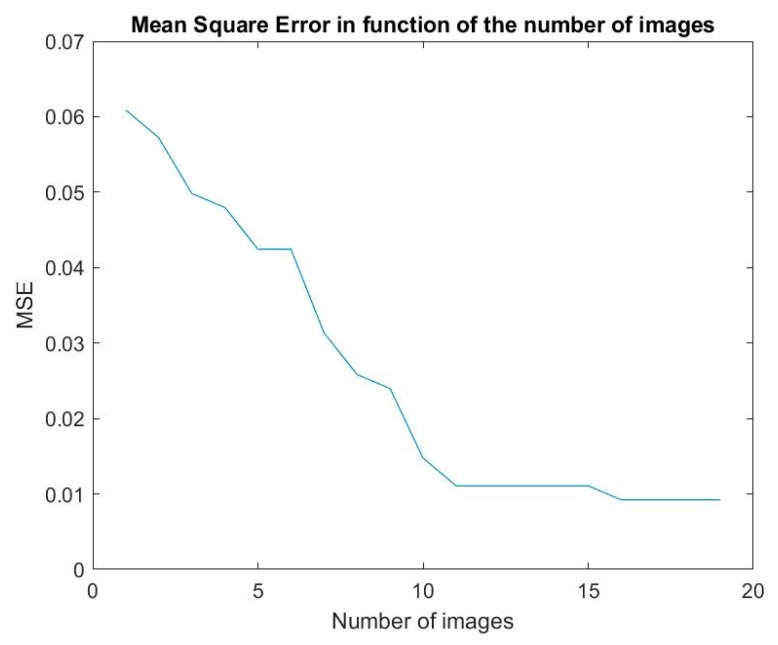
The mean square error (MSE) versus the number of images in the training set.

In Figure 6, we plot the MSE calculated with different numbers of images in the training set. The training set was made of 14 × (2*m* + 1) images, where *m* is the number of images used from either side of the image centered on the face (horizontal shift) for all 14 persons. 2*m* + 1 is the total number of images used for one person: One for the centered face, m images with horizontal shift on the right, and m images with horizontal shift on the left. We observe that when *m* is in the range 1–11, the MSE decreases linearly, but for larger values of *m*, the MSE is almost constant at a value of 1%, indicating the good level of performance of our method. Thus, it is not necessary to build a learning process of the algorithm on the whole database. This avoids the so-called problem of overfitting.

**Figure 7:**
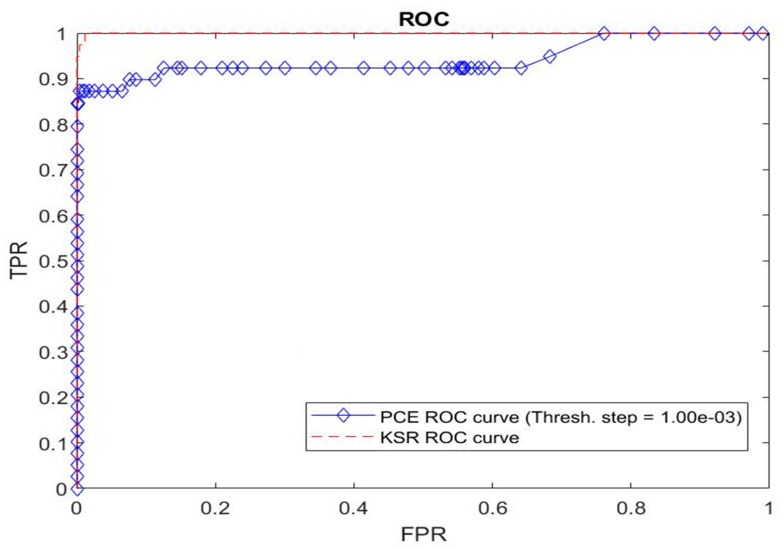
ROC curves on the testing set of our method (KSR) and peak-to-correlation energy (PCE) criterion. Plot of true positive rate (TPR) vs false positive rate (FPR).

Figure 7 shows the Receiver Operating Characteristic (ROC) curve to check the ability of this algorithm to classify person 0 and the others correctly. The area under the curve is 0.9996. In order to compare with existing methods, we plotted the ROC curve with the peak-to-correlation energy (PCE) criterion for the same data. We observe that our method leads to much better results. We then have a near perfect classifier which clearly outperforms the standard algorithm using the PCE criterion.

### 6.4. Computation Time

**Figure 8:**
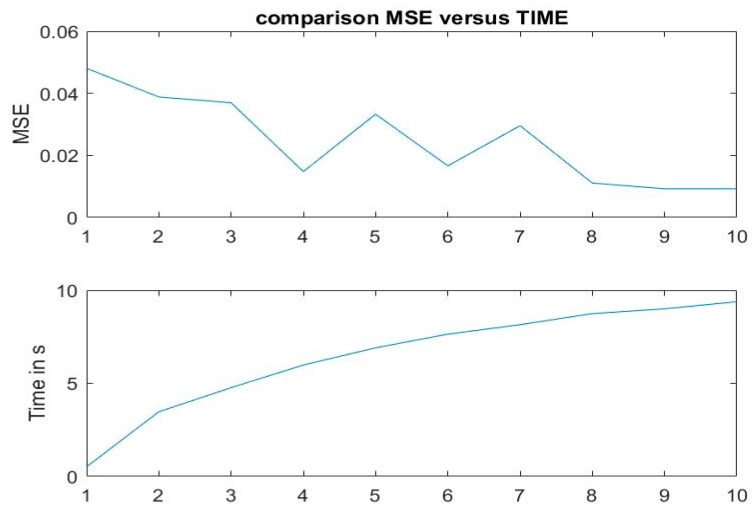
MSE and corresponding running times (in seconds) obtained by reducing the image size in the set of images.

With a training set of 541 images, the running time of the algorithm was 11 s. We now show how to improve the computation time by reducing the image size. One drawback of this method is that the performance of the algorithm can be affected by reducing the size of the correlation plane. The selection of one over two pixels was first considered, then an increase by considering 2 out of 3 was considered, then 3 out of 4 and so on, until 10 out of 11. When selecting 1 out of 2 pixels, it takes almost 0.5 s to perform the algorithm with a MSE of 4.81%. For 4 out of 5 pixels, the MSE is close to 1.5% and the running time is less than 6 s (Figure 8). In Figure 8, the top plot shows the MSE according to the number of pixels used and the bottom plot shows the corresponding running time. As the number of pixels is increased, the MSE decreases due to the loss of information in making the correlation planes. But the computation time increases with the number of pixels used. A good compromise is to use 4 out of 5 pixels. This yields to a running time less than 6 s

### 6.5. Simulations for a Second Series of Faces from the PHPID Database

**Figure 9:**
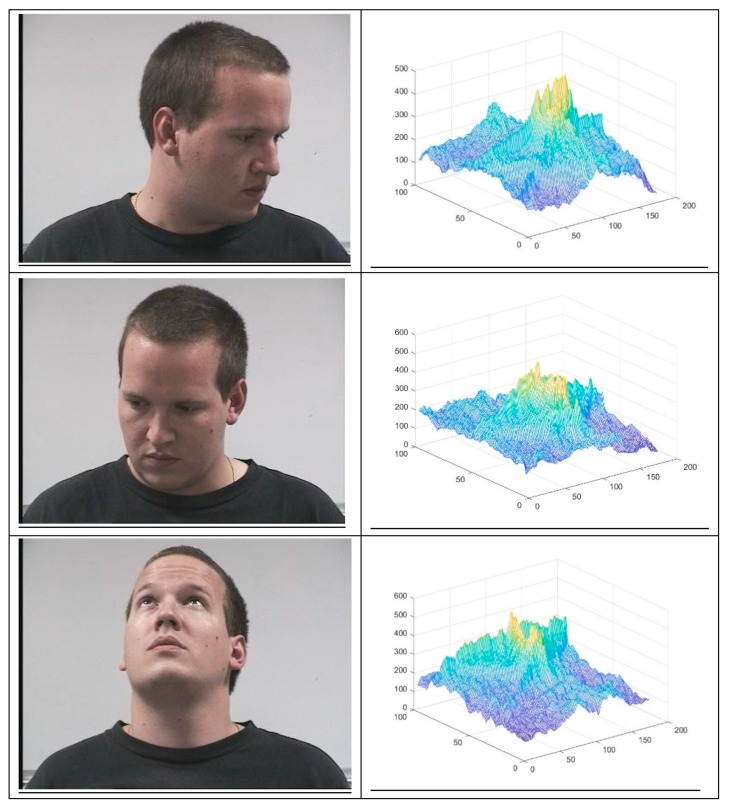

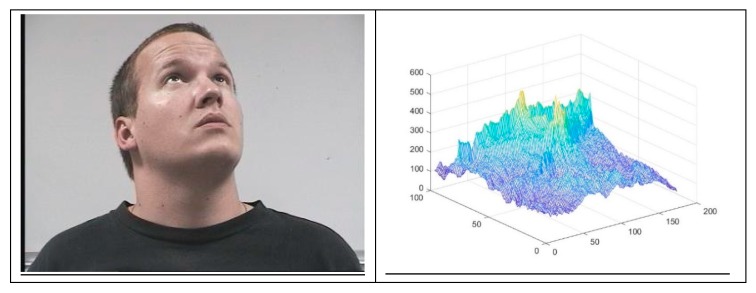
Images and correlation planes well recognized by our method (KSR) and badly recognized by PCE.

Consider a second series of faces from the PHPID database. These simulations differ from the previous database, since the clothes and haircuts are different and the persons can wear glasses. Here, there are 93 images of person 0 with different poses. For the training set, we use the 541 images coming from the series 1 so that we have 39 images of person 0. We find an error rate of 12.9%. For 93 images, only 12 were not detected. To illustrate the performance of our procedure, Figure 9 shows 4 images and their corresponding correlation planes. The 4 images have been well recognized by our method, where the PCE method gives an error. The first column represents the 4 images and the second column represents the corresponding correlation planes with the image of reference of person 0.

**Figure 10:**
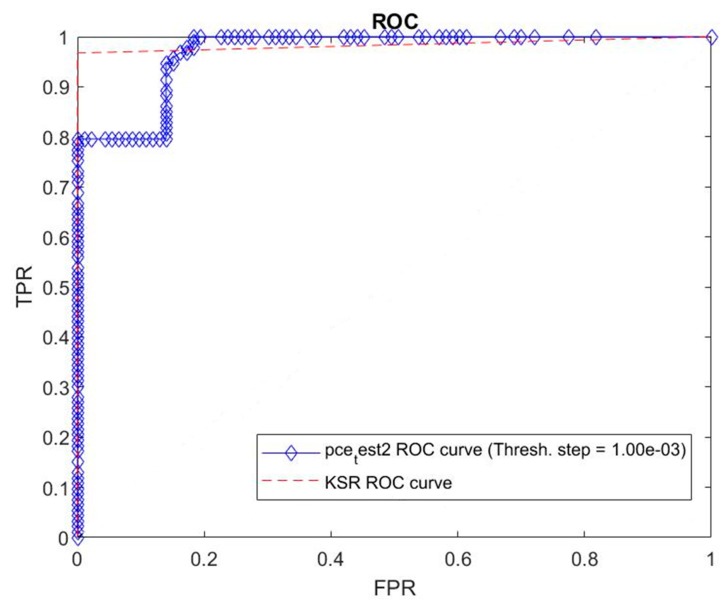
Same as Figure 7 for the second series of faces from the PHPID database.

In order to compare our results with those obtained using the PCE criterion, we provide a comparison of the ROC curves in Figure 10. We conclude that our method is significantly better than the method using the PCE criterion. To make this plot, we used 93 images of two persons from the second series of faces from the PHPID database as the testing set. The training set comprised 541 images (from which, there were 39 images of person 0) from the first series of faces from the PHPID database.

## 7. Conclusions

We presented an innovative method specially designed to enhance the decision performance for face recognition applications. This approach is based on a classification algorithm by means of nonparametric estimation of the regression function, which defines the probability of recognition. A two-step procedure was developed, considering first the construction of the correlation planes and then the decision-making based on a kernel smoothing regression algorithm. The results and their discussion show that this easy and fast to implement algorithm performs very well using the PHPID dataset. Our results are useful because we reach a very good level of recognition, namely less than 1% of MSE. This method can be extended in parametrically modelling correlation planes.
